# Gait Characteristic Analysis and Identification Based on the iPhone's Accelerometer and Gyrometer

**DOI:** 10.3390/s140917037

**Published:** 2014-09-12

**Authors:** Bing Sun, Yang Wang, Jacob Banda

**Affiliations:** 1 School of Electronics and Information Engineering, Beihang University, 37 Xueyuan Road Haidian District, Beijing 100191, China; 2 School of Electrical and Electronic Engineering, Nanyang Technological University, 50 Nanyang Avenue, 639798, Singapore; E-Mail: ywang02@outlook.com; 3 Department of Mathematics, the University of Texas-Pan American, 1201 West University Drive, Edinburg, TX 78539, USA; E-Mail: jnbanda@broncs.utpa.edu

**Keywords:** gait, iPhone, accelerometer, identification, weighted voting

## Abstract

Gait identification is a valuable approach to identify humans at a distance. In this paper, gait characteristics are analyzed based on an iPhone's accelerometer and gyrometer, and a new approach is proposed for gait identification. Specifically, gait datasets are collected by the triaxial accelerometer and gyrometer embedded in an iPhone. Then, the datasets are processed to extract gait characteristic parameters which include gait frequency, symmetry coefficient, dynamic range and similarity coefficient of characteristic curves. Finally, a weighted voting scheme dependent upon the gait characteristic parameters is proposed for gait identification. Four experiments are implemented to validate the proposed scheme. The attitude and acceleration solutions are verified by simulation. Then the gait characteristics are analyzed by comparing two sets of actual data, and the performance of the weighted voting identification scheme is verified by 40 datasets of 10 subjects.

## 1. Introduction

Gait refers to an idiosyncratic feature of a person, that is determined by an individual's weight, limb length, posture combined with characteristic motion [1] and so on. Also gait may reflect the health condition of humans [2,3], such as symptoms of Cerebral Palsy and Parkinson's disease. Hence, gait can be used as a biometric measure to recognize known persons and classify unknown subjects [1]. Gait identification allows a nonintrusive way of data collection from a distance. This procedure can provide supplemental information for the traditional approaches (e.g., fingerprint, iris, face, voice) in security and usability applications, thereby enhancing or complimenting them.

At present, gait analysis can be classified according to two different approaches: non-wearable sensors (NWS) based and wearable sensors (WS) based [4]. NWS systems usually require optical sensors [5–8] or pressure sensors on the floor [9]. Gait features are extracted by processing image or video data. In the security surveillance field, this approach can effectively narrow the range of subjects in gait database and plays an important role. WS systems are based on motion-recording sensors which are attached to moving subjects [10–16]. Motion-record sensors (e.g., accelerometers, gyrometers, force sensors, bend sensors, and so on) are usually attached to various parts of the body such as the ankle, hip and waist [12,16]. Then, gait signatures are extracted by analyzing the recorded signals in both time and frequency domains. With the application of a Microelectromechanical System (MEMS), a device such as an Inertial Measurement Unit (IMU) is easily inserted to shoes, gloves, watches and other wearable attire. The prospect of applying wearable sensors to gait analysis is discussed in [12–15].

Smart mobile phones, nowadays widely equipped with MEMS accelerometers and gyrometers to determine device orientation, provide developers and users with a rich and easily accessible platform for exploration. Plenty of application programs are developed based on smartphone sensors. An iPhone is used as a wireless accelerometer sensor for Parkinson's disease tremor analysis in [17]. The feasibility of gait identification using an iPhone's accelerometer is discussed in [18]. However, current study of gait analysis based on fixed smartphone sensors mostly use data including gravitational acceleration without considering the effects of attitude change. Our goal is mainly to analyze gait characteristics and identification with an unfixed iPhone in pant pockets instead of professional sensors and laboratory conditions. Under this case, attitude of the iPhone should be considered to minimize the effects of attitude change and gravitational acceleration. After extracting some gait information, a voting scheme is investigated for gait identification.

This paper is organized as follows. In Section 2, pre-processing methods for iPhone inertial sensor data are introduced. In Section 3, gait characteristics are analyzed and methods are studied to extract gait parameters from accelerometer and gyrometer data. In Section 4, a weighted voting scheme based on gait signatures is proposed for gait identification. In Section 5, validating experiments are conducted for gait analysis and identification using both simulated data and actually collected data.

## 2. Pre-Processing of iPhone Measurement Data

Several kinds of sensors are integrated in an iPhone like a GPS sensor, accelerometer, gyrometer, magnetometer, and so on. Theoretically, the motion of an iPhone (including its acceleration, velocity, and position) can be solved based on the sensor data. However, according to experiments, the precision of iPhone sensors [19] can hardly support accurate solutions of device velocity and position, especially for short-distance measurements. According to the operating principles of iPhone accelerometers, the collected data is in its own inertial coordinate system, and has gravitational acceleration included. During the collection of an individual's gait data, the attitude of an iPhone is changing so it is not an accurate method to directly process the measured acceleration data. In this section, a pre-processing approach is studied to get the linear acceleration, in which the gravitational acceleration is not included. This is achieved by defining a reference coordinate system with its Z-axis corresponding to the opposite direction of gravity.

### 2.1. Inertial Data Calibration

**Figure 1.**
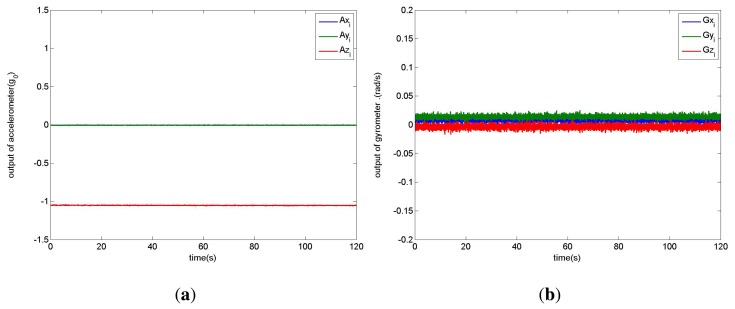
The direct outputs of triaxial (**a**) accelerometer and (**b**) gyrometer.

Although the inertial sensors are calibrated by the manufacturers, there are still some systematic errors for applications. Figure 1 illustrates the direct outputs of the triaxial accelerometer and gyrometer, which are collected when the iPhone is placed on a horizontal surface for 120 s. It is obvious that the values of z-axis acceleration deviate from the theoretical value −1.0 *g_0_, where *g*_0_ = 9.81 m/s^2^ is the gravitational acceleration constant. A similar situation occurs for the gyrometer. So the calibration of inertial data is needed.

#### 2.1.1. Static State Judgement

To determine the initial attitude of an iPhone, an approximate static state is required. If the variance of the complex acceleration amplitude is below a threshold in a short period (e.g., 1 s), the iPhone can be regarded as static or at uniform motion in a straight line. Due to ups and downs during walking, uniform motion is almost impossible. In a static state, the measured acceleration data corresponds to the gravity only.

#### 2.1.2. Acceleration Bias

There may be systematic errors in accelerometers because of device differences, device aging, or even occasional damage to the device. The systematic error is coupled with motion error and gravitational acceleration, and leads to the acceleration bias in measurements. The acceleration bias can be estimated by recording triaxial accelerations under static state and calculating their mean values. Thus in the processing of gait data, acceleration bias can be eliminated by directly subtracting the estimated bias from triaxial acceleration data. In Figure 1a, the main bias comes from z-axis, which is about −0.05 * *g*_0_.

#### 2.1.3. Gyrometer Bias and Drift

The theoretical outputs of triaxial gyrometer should be zeros in a static state. We also calculate their mean values to be the systematic biases and subtract these biases from triaxial gyrometer data. Usually the biggest problem for a gyrometer is its drift during long time measurement. However, according to Figure 1b we can see the drift in 120 s is almost zero. Therefore in a short time period the drift can be neglected in gait measurement, which is usually sampled in less than 20 s intervals.

### 2.2. Attitude during Motion

**Figure 2.**
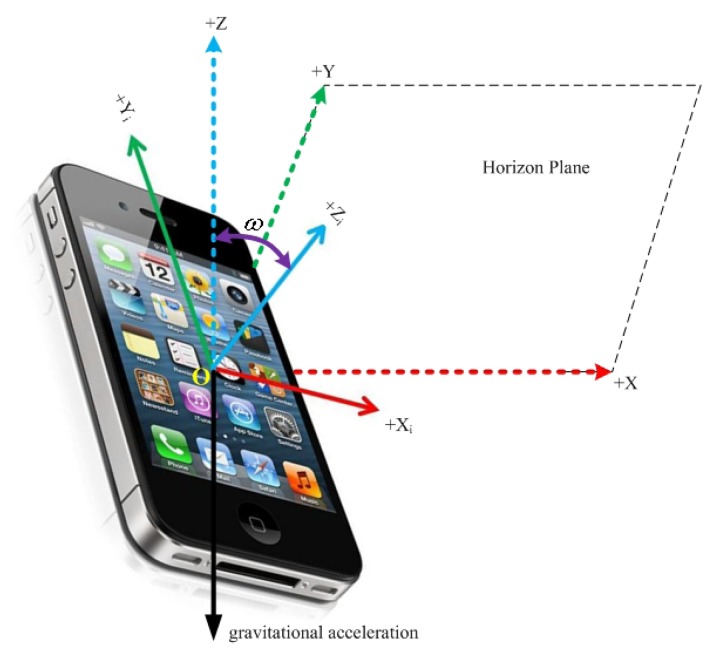
iPhone inertial coordinate system and reference coordinate system.

To study the attitude of the iPhone sensor during motion, a reference coordinate system is defined based on gravity and the initial attitude of the iPhone sensor. In this paper, attitude is described by quaternion, which can be calculated via the combination of acceleration and gyrometer data. Then the coordinate transformation matrix is solved. The specific process is as follows:

(1) To define the reference coordinate system:
  The iPhone sensor has its own inertial coordinate system [*O*, *X*,*_i_*,*Y_i_*,*Z_i_*], as shown in Figure 2. Define *Z*-axis of the reference coordinate system opposite to the gravity vector, *ω* as the angle between the *Z_i_*-axis and *Z*-axis, and a unit vector *C* =(*c*_1_,*c*_2_,*c*_3_) which is vertical to both *Z_i_*- and *Z*-axis as the rotation axis. For the initial attitude of iPhone sensor, the triaxial accelerations are *A*_0_ =(*a_x_*_0_,*a_y_*_0_,*a_z_*_0_)*^T^* in iPhone inertial coordinate system, and the gravitational acceleration is *B*_0_ = (0, 0, −*g*_0_)*^T^* in reference coordinate system, then *ω* and *C* can be obtained as $\omega =\mathrm{acos}\left(\frac{A_{0}.B_{0}}{\left|A_{0}\right|.\left|B_{0}\right|}\right)$, $C=\frac{A_{0}\times B_{0}}{\left|A_{0}\times B_{0}\right|}$. After a rotation around *C* by an angle of *ω*, the *Z_i_*-axis is rotated to *Z*-axis, and the *X_i_*-axis and *Y_i_*-axis are rotated to the horizontal plane, in which they are now defined as *X*-axis and *Y*-axis. [*O*, *X*, *Y*, *Z*] is the reference coordinate system for further analysis.
  According to the quaternion as rotations [20,21], the initial quaternion *Q*_0_ = [*w*_0_, *x*_0_, *y*_0_, *z*_0_]*^T^* is solved as:
  (1) $$w_{0}=\cos\left(\frac{\arccos\omega }{2}\right)$$
  (2) $$x_{0}=c_{1}\sin\omega$$
  (3) $$y_{0}=c_{2}\sin\omega$$
  (4) $$z_{0}=c_{3}\sin\omega$$
  It means one time rotating around unit vector C=(*c*_1_,*c*_2_,*c*_3_) by ω transforms the iPhone inertial coordinate to the defined reference coordinate.
  In a further step, the three Euler angles of the iPhone can be solved based on quaternion. However, the values of Euler angles depend on their definition and rotations [22]. In this paper, quaternion is adopted for iPhone solution of attitude.
(2) Update quaternion:
  To update quaternion *Q_i_* = [*w_i_*, *x_i_*, *z_i_*]*^T^* with combined acceleration data $A_{i}={\left(a_{x}^{i},a_{y}^{i},a_{z}^{i}\right)}^{T}$ and gyrometer data $g_{i}={\left(g_{x}^{i},g_{y}^{i},g_{z}^{i}\right)}^{T}$ using the Fourth-order Runge-Kutta Algorithm [23], the specific process is as follows:
  (5) $$Q_{i+1}=Q_{i}+\frac{T}{6}\left(K_{1}+2K_{2}+2K_{3}+K_{4}\right)$$
  (6) $$K_{1}=\frac{1}{2}Q_{i}⋆g_{i}$$
  (7) $$K_{2}=\frac{1}{2}\left[Q_{i}+\frac{K_{1}}{2}T\right]⋆g_{i}+\frac{1}{2}$$
  (8) $$K_{3}=\frac{1}{2}\left[Q_{i}+\frac{K_{2}}{2}T\right]⋆g_{i}+\frac{1}{2}$$
  (9) $$K_{4}=\frac{1}{2}\left[Q_{i}+K_{3}T\right]⋆g_{i}+1$$
  where *Q_i_* ⋆ g*_i_* refers to quaternion product,
  (10) $$Q_{i}*g_{i}=\left[\begin{array}{ccc} -x^{i} & -y^{i} & -z^{i}\\ \omega^{i} & -z^{i} & y^{i}\\ z^{i} & \omega^{i} & -x^{i}\\ -y^{i} & x^{i} & \omega^{i} \end{array}\right]\;\left[\begin{array}{c} g_{x}^{i}\\ g_{y}^{i}\\ g_{z}^{i} \end{array}\right]$$
  and $g_{i+\frac{1}{2}}=\frac{1}{2}\left(g_{i}+g_{i+1}\right)$.
(3) Calculate coordinate transformation matrix:
  To find the coordinate transformation matrix $T_{om}^{i}$ for *t* = *i*, according to real-time quaternion *Q_i_* =[*w_i_*, *x_i_*, *y_i_*, *z_i_*]*^T^*. The coordinate transformation matrix [22] is
  (11) $$T_{om}^{i}=\left[\begin{array}{ccc} 1-2\left(y_{i}^{2}+z_{i}^{2}\right) & 2\left(x_{i}y_{i}-\omega_{i}z_{i}\right) & 2\left(x_{i}z_{i}-\omega_{i}y_{i}\right)\\ 2\left(x_{i}y_{i}-\omega_{i}z_{i}\right) & 1-2\left(x_{i}^{2}-z_{i}^{2}\right) & 2\left(y_{i}z_{i}-\omega_{i}x_{i}\right)\\ 2\left(x_{i}z_{i}-\omega_{i}y_{i}\right) & 2\left(y_{i}z_{i}-\omega_{i}x_{i}\right) & 1-2\left(x_{i}^{2}-y_{i}^{2}\right) \end{array}\right]$$

### 2.3. Linear Acceleration Solution

Gravitational acceleration can be eliminated to get the linear acceleration ${\left(l_{x}^{i},l_{y}^{i},l_{z}^{i}\right)}^{T}$ using the coordinate transformation matrix, described as follows

(12) $$\left[\begin{array}{c} l_{x}^{i}\\ l_{y}^{i}\\ l_{z}^{i} \end{array}\right]=T_{om}^{i}\left[\begin{array}{c} a_{x}^{i}\\ a_{y}^{i}\\ a_{z}^{i} \end{array}\right]-\left[\begin{array}{c} 0\\ 0\\ -g_{0} \end{array}\right]$$

The first term of the right side of Equation (12) is the total acceleration in reference coordinate system. After subtracting the gravitational acceleration in the same reference coordinate system, we can get the linear acceleration. Since the two components $l_{x}^{i}$ and $l_{y}^{i}$ correspond to the horizon direction of motion, the following gait characteristic analysis is based mainly on the component of linear acceleration $l_{z}^{i}$.

## 3. Gait Characteristic Analysis

In this section, we discuss four gait characteristic parameters: gait frequency, symmetry coefficient, dynamic range and similarity degree between characteristic curves. The location-related parameters are not considered because the integration of acceleration will generate accumulation error.

### 3.1. Gait Frequency

**Figure 3.**
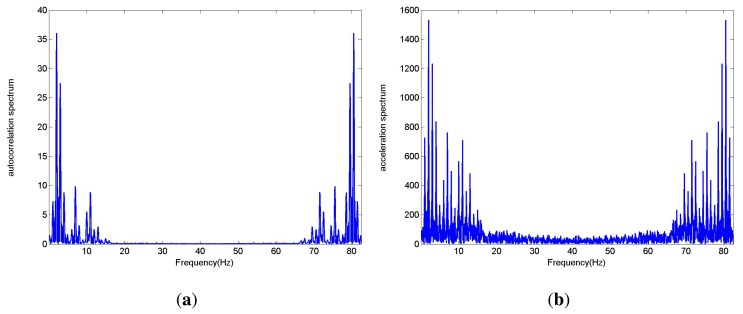
Acceleration spectrum: (**a**) FFT of autocorrelation; (**b**) FFT of acceleration.

Gait frequency is the most basic and important parameter in gait characteristics analysis. Due to the quasi-periodic characteristic of paces during walking, gait frequency can be extracted by the characteristic of the acceleration signal in the frequency domain. After FFT of the acceleration signal, the maximum frequency, excluding DC component, is not always the gait frequency. An improved method is presented in this paper. The autocorrelation of the acceleration signal is calculated and converted to frequency domain. Then the maximum frequency, excluding zero frequency component, equals to gait frequency. Figure 3 shows the spectrum of acceleration signals and their autocorrelation. Interpolation processing can greatly improve the accuracy of gait frequency while calculating correlation coefficient.

According to the Figure 3, the spectrum lines of the autocorrelation function are more focused than those of the acceleration. Thus the autocorrelation function of acceleration is usually less affected by harmonic components and easier to extract gait frequency.

### 3.2. Symmetry Coefficient

**Figure 4.**
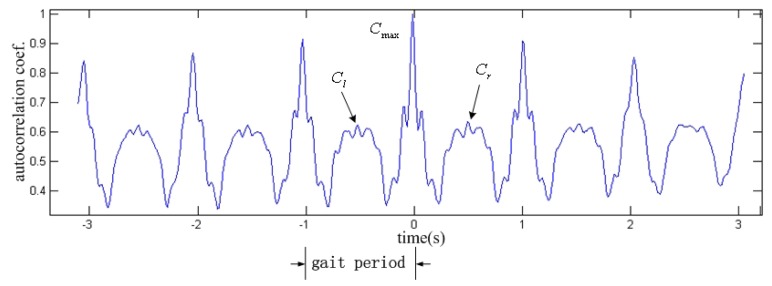
Schematic definition of gait symmetry. The blue line is the autocorrelation curve of acceleration.

Some literature has defined the gait symmetry corresponding their own dataset [24]. In this paper, we define the symmetry coefficient *S_i_* of gait according to autocorrelation of acceleration signal, shown in Figure 4.

(13) $$S_{i}=\frac{1}{2}\left(\frac{C_{l}}{C_{\max}}+\frac{C_{r}}{C_{\max}}\right)$$

where *C*_max_ is the maximum of the autocorrelation of acceleration, *C_l_* and *C_r_* are the maximum values of the points located on the left and right of the peak position half gait period away, respectively.

Two possible causes may lead to asymmetry of the acceleration signal: (1) inconsistent gait, such as the gait of patients with unilateral actions and other obstacles; (2) sensors are not strictly centered placed. The first case is not considered in this paper, and all subsequent tests are based on almost symmetrical gait. We will discuss the effect of sensor's positions in Section 5.

### 3.3. Dynamic Range

The dynamic range of acceleration can reflect a gait characteristic in some aspects. In this paper, the difference between the maximum and minimum values of Z-linear acceleration is defined as dynamic range estimation.

(14) $$D=\max\left(l_{z}^{i}\right)-\min\left(l_{z}^{i}\right)$$

Usually, a few aspects including the speed of walking, pace length, noise and so on will affect the dynamic range. It is reasonable that dynamic range should has less contribution during gait identification.

### 3.4. Similarity Degree between Characteristic Curves

For typical gait acceleration, the characteristic curve can be extracted according to its fluctuation characteristics, that is the time-amplitude sequence. This sequence can be used as an important basis for the subsequent gait matching and identification. By comparing the characteristic curve with each characteristic curve in database, a high similarity degree indicates the two curves correspond to the same people with a high probability, and it is helpful in gait identification.

To accurately estimate the similarity degree between two characteristic curves, scale transformation and panning of the curves have to be taken into consideration. Even for the same subject, walking speed and magnitude may have subtle variations under different test conditions. The discrete acceleration data cannot be directly used for similarity comparison. Although a one-dimensional invariant matrix method can be used theoretically, the experimental performance is not ideal. In this paper, the similarity degree between characteristic curves is calculated as follows:

1. apply interpolation processing, according to the gait frequencies, to eliminate the effects of scale differences.
2. find the peak positions of two curves as reference, and make a rough alignment of the vectors. The aligned acceleration characteristic curves are signed as *l*_1_, *l*_2_.
3. calculate the similarity coefficient, which is used to describe similarity degree, based on the normalized dot product of two vectors. Moreover, to eliminate noise affect in characteristic curves, we translate one vector within one pace period, which corresponds to N samples, then calculate the normalized dot product of the vectors, and finally select the maximum normal product as the similarity coefficient *C*.

(15) $$C=\underset{0\leq m<N}{\max}\frac{\overset{N}{\underset{i=1}{\text{∑}}}\left[l_{1}\left(i+m\right).l_{2}\left(i\right)\right]}{\sqrt{\overset{N}{\underset{i=1}{\text{∑}}}l_{1}^{2}\left(i+m\right).\overset{N}{\underset{i=1}{\text{∑}}}l_{2}^{2}\left(i\right)}}$$

## 4. Gait Identification

In the previous section, several gait characteristic parameters including gait frequency, symmetry coefficient, dynamic range and similarity coefficient are discussed. Based on these typical parameters, a weighted voting scheme is proposed for gait identification. The specific scheme is as follows:

1. calculate gait frequency *F_i_*, symmetry coefficient *S_i_*, dynamic range *D_i_* for all the characteristic curves in the database one by one. Suppose the number of the curves in database is *M*, *i* =1, 2, …, *M*.
2. calculate gait frequency *F* , symmetry coefficient *S*, dynamic range *D* of the linear acceleration from the measured data.
3. calculate the similarity coefficient *C_i_* between the characteristic curves of linear acceleration and samples in the database.
4. the weighted voting process is as follows:
  (a) sort the absolute error between *F* and *F_i_* in increasing order. We vote the first term 1, the second term 2, and so on. The number of votes of gait frequency is denoted as $V_{i}^{1}$.
  (b) sort the absolute error between *S* and *S_i_* in increasing order. We vote the first term 1, the second term 2, and so on. The number of votes of symmetry coefficient is denoted as $V_{i}^{2}$.
  (c) sort the absolute error between *D* and *D_i_* in increasing order. We vote the first term 1, the second term 2, and so on. The number of votes of dynamic range is denoted as $V_{i}^{3}$.
  (d) sort *C_i_* in decreasing order. We vote the first term 1, the second term 2, and so on. The number of votes of similarity coefficient is denoted as $V_{i}^{4}$.
  (e) sum the weighted number of votes for each sample in the database.
    (16) $$V_{i}=w_{1}V_{i}^{1}+w_{2}V_{i}^{2}+w_{3}V_{i}^{3}+w_{4}V_{i}^{4},i=1,2,\ldots ,M$$
    where *W* =[*w*_1_,*w*_2_,*w*_3_,*w*_4_] is the weighted coefficient vector.
  (f) vote judgment. The sample in the database that has the minimum weighted vote summation matches the current measured data, and we can accomplish gait identification within the given database.

The weighted coefficient can be chosen by further experimental data. The similarity coefficient of characteristic curves is of the most importance for gait identification, followed by gait frequency. The symmetry coefficient has a close relationship with the iPhone's placement. The dynamic range is greatly affected by noise. Therefore, the weighted coefficients are set as *W* =[2, 2, 1, *w*_4_] in this paper, where

(17) $$w_{4}=\{\begin{array}{ll} 8 & C_{max}\in \left[0.9,1\right]\\ 4 & C_{max}\in [0.8,0.9)\\ 2 & C_{max}\in [0.7,0.8)\\ 1 & C_{max}\in [0,0.7) \end{array}\;,C_{\max}=\underset{i\in \left[1,M\right]}{max}\left\{C_{i}\right\}$$

## 5. Experiments

Four experiments are conducted to validate the methods proposed in this paper: (1) simulation experiment to verify the solutions of attitude and linear acceleration; (2) comparison experiment of the same subject walking on cement pavement and on grass; (3) comparison experiment of three different placements of iPhones; (4) gait identification experiment with 10 subjects and 40 sets of data.

**Figure 5.**
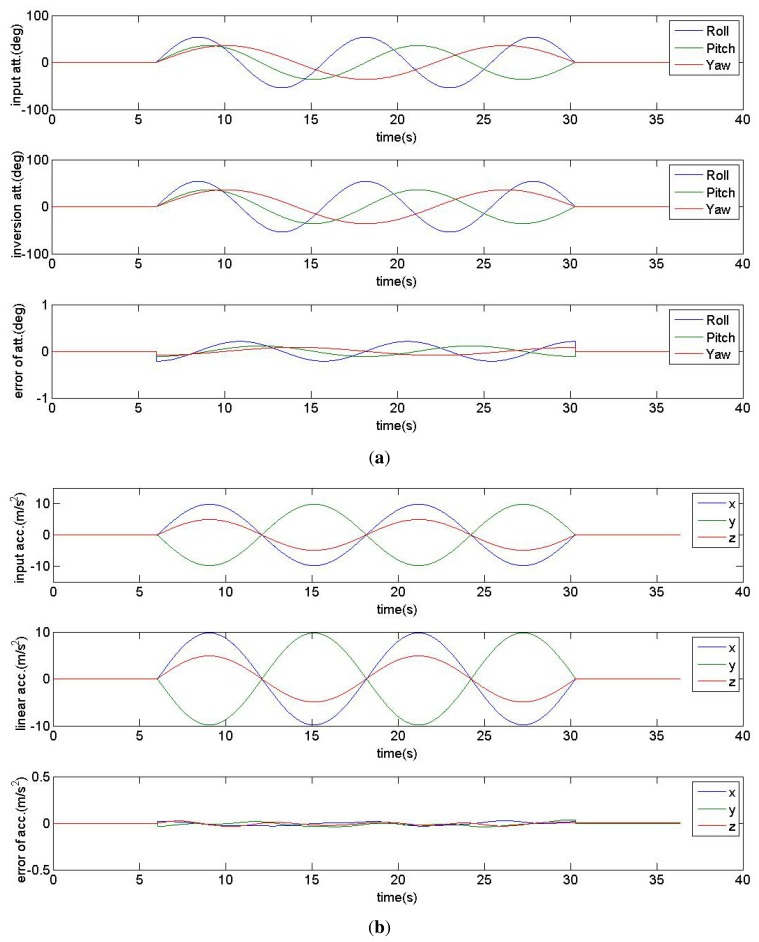
Inversion results of simulated data. The top, middle and bottom curves in (**a**) are the input data, inversion data and errors of the roll, pitch and roll attitudes, respectively. The top, middle and bottom curves in (**b**) are the input data, inversion data and errors of the x-, y-and z- linear accelerations, respectively.

In the first experiment, the sinusoidal linear acceleration and attitude data are simulated. The attitude angles (yaw, pitch and roll) are defined by Tait-Bryan angles formalism with rotation sequence z-y-x [25]. Then the triaxial accelerometer and gyrometer data in iPhone's inertial coordinate system is generated as the input of the proposed method in Section 2. After calculation and comparison, the inversion linear acceleration and attitude data, and the errors are shown in Figure 5.

According to simulation results, the inversion errors of attitude are less than 1°, and the inversion errors of acceleration are less than 0.1 m/s^2^.

**Figure 6.**
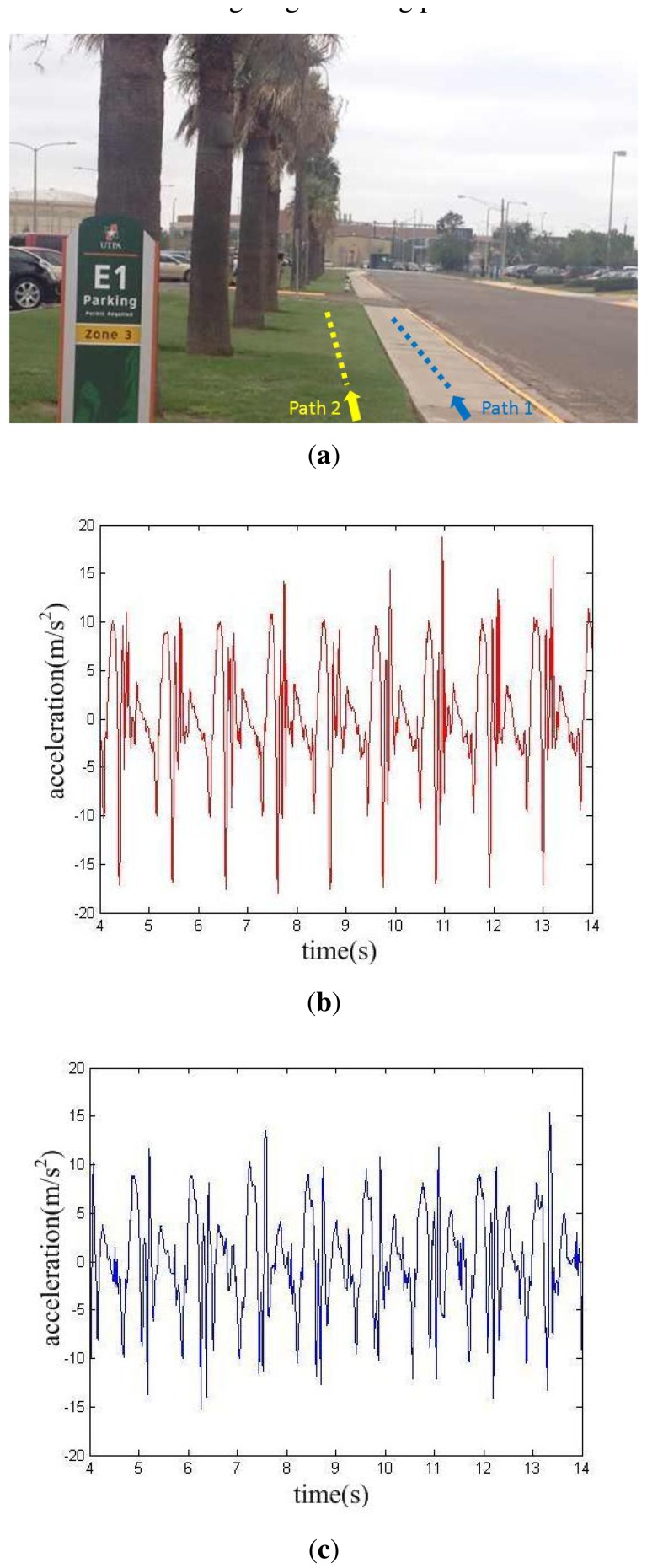
Comparison experiment with different ground conditions. (**a**) Test conditions and the two test paths. (**b**) Linear acceleration data of walking on cement pavement along path 1. (**c**) Linear acceleration data of walking on grass along path 2.

In the second experiment, the subject walked on cement pavement and grass, respectively, with the same iPhone placed in the right pant pocket. The characteristic curves of recorded acceleration data are shown in Figure 6.

At least two differences between the two datasets can be seen in Figure 6.

1. The data recorded on cement pavement is nearly 10 cycles, while the data recorded on grass is less than 9 cycles, both recorded in 10 s. They are consistent with the gait periods calculated by gait frequency analysis, which are 1.070 s and 1.174 s, respectively. This is due to the fact that it's more difficult to walk on soft surfaces like grass than on cement pavement.
2. The dynamic range of linear acceleration recorded while walking on cement pavement is a little larger than that on grass, and it can be explained by the buffer caused by grass.

**Figure 7.**
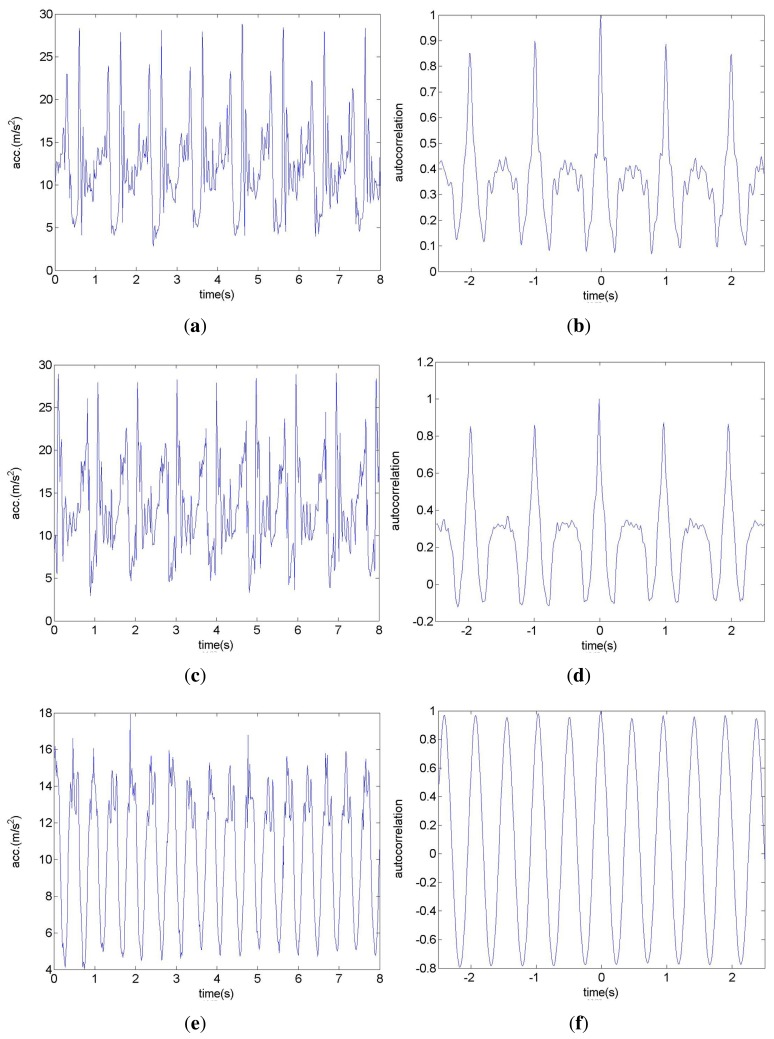
Linear acceleration and autocorrelation curves. (**a**) and (**b**) correspond to the data collected by the iPhone in left pant pocket. (**c**) and (**d**) correspond to the data collected by the iPhone in right pant pocket. (**e**) and (**f**) correspond to the data collected by the iPhone in the center of the back waist area.

In the third experiment, three iPhones are placed in three different positions: the left pant pocket, the right pant pocket and the center of the back waist area of the subject. The calculated linear accelerations and autocorrelation curves are shown in Figure 7.

According to Figure 7a,c,e, the linear acceleration signal reflects preferable quasi-periodicity. Moreover, the curves in Figure 7a,c are very similar and differ mainly in half gait period in time. The third data shows good symmetry among each single step, and the frequency is twice of the above two curves. According to Figure 7b,d, the accelerations corresponding to the left and right pace are asymmetrical, obviously due to the off-center displacement. Furthermore, the symmetry coefficients are only about 0.4. Figure 7f describes the third case, when the iPhone was placed in the center of the back waist. The left and right paces cause similar linear accelerations, and the symmetry coefficient is more than 0.9. The definition of symmetry coefficient is also validated. Moreover, the dynamic ranges of Figure 7a,b are greater than that in Figure 7c as a result of the up and down leg motion versus the body motion within the same pace period.

**Figure 8.**
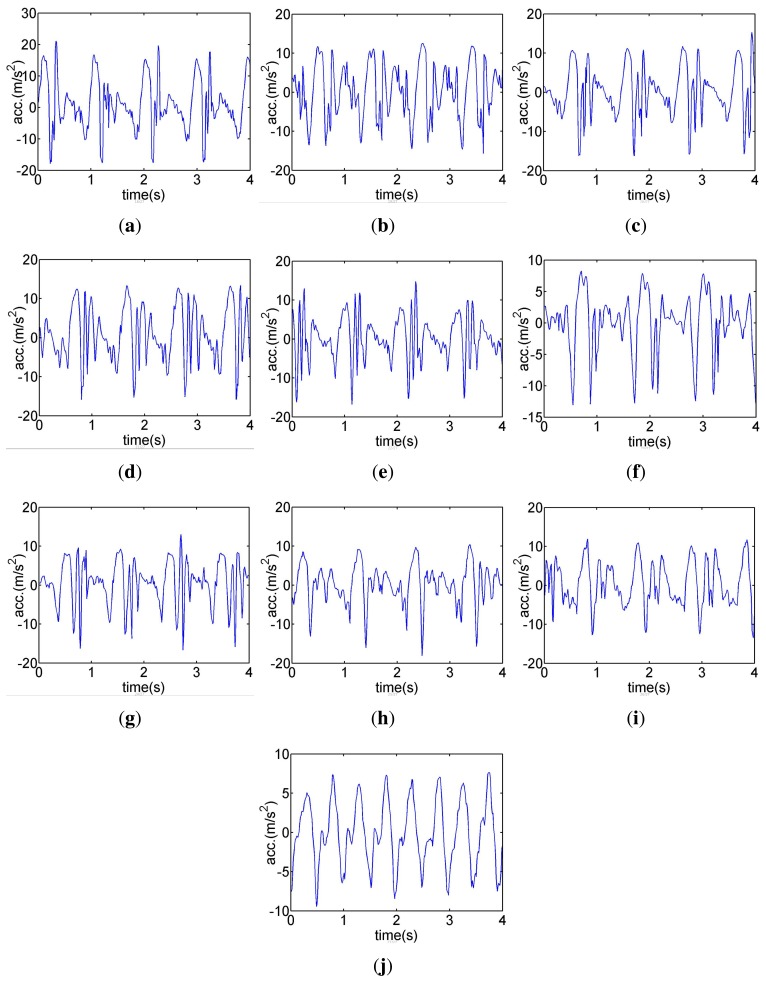
Characteristics curves of ten tested persons as the database.

In the fourth experiment, a single iPhone is used to record 4 sets of gait data for each of the 10 tested subjects. The phone is placed in their left and right pant pockets twice, respectively. Then one set of data is selected for each subject in order to make the gait database by calculating characteristic parameters and extracting characteristic curves, shown in Table 1 and Figure 8.

The remaining 30 sets of recorded data are used as measured data to compare with 10 samples in the database. The voting results of 10 groups of measured data is listed in Table 2. We calculate the weighted votes for measured data M1∼M10 according to the method described in Section 4. For example, data M1 yields gait frequency *F_i_* = 1.039 Hz, symmetry coefficient *S*_1_ = 0.554, and dynamic range *D*_1_ = 25.87 m/s^2^ . The similarity coefficients between M1 and D1∼D10 are [*C*_1_*,C*_2_*,C*_3_*,C*_4_*,C*_5_*,C*_6_*,C*_7_*,C*_8_*,C*_9_*,C*_10_]=[0.876, 0.773, 0.667, 0.762, 0.793, 0.498, 0.614, 0.735, 0.794, 0.575]. After comparing the above parameters with Table 1 and sorting the absolute errors in increasing order and similarity coefficients in decreasing order, we can get the votes of gait frequency $\left[V_{1}^{1},V_{2}^{1},V_{3}^{1},V_{4}^{1},V_{5}^{1},V_{6}^{1},V_{7}^{1},V_{8}^{1},V_{9}^{1},V_{10}^{1}]=[2,1,7,4,9,10,5,8,6,3\right]$, the votes of symmetry coefficient $\left[V_{1}^{2},V_{2}^{2},V_{3}^{2},V_{4}^{2},V_{5}^{2},V_{6}^{2},V_{7}^{2},V_{8}^{2},V_{9}^{2},V_{10}^{2}\right]=[3,1,2,9,8,5,7,6,4,10]$, the votes of dynamic range $\left[V_{1}^{3},V_{2}^{3},V_{3}^{3},V_{4}^{3},V_{5}^{3},V_{6}^{3},V_{7}^{3},V_{8}^{3},V_{9}^{3},V_{10}^{3}\right]=[3,6,2,7,4,1,5,9,8,10]$, and the votes of similarity coefficient $\left[V_{1}^{4},V_{2}^{4},V_{3}^{4},V_{4}^{4},V_{5}^{4},V_{6}^{4},V_{7}^{4},V_{8}^{4},V_{9}^{4},V_{10}^{4}\right]=[1,4,7,5,3,10,8,6,2,9]$. Following multiplication by *W* = [2, 2, 1*,ω*_4_], where *ω*_4_ = 4 is calculated using Equation (17), the summation votes, using Equation (16), equal to [17, 26, 48, 53, 50, 71, 61, 61, 36, 72] and are displayed in the first row in Table 2. The minimum of this row is 17, which corresponds to data D1. Because datasets D1 and M1 are collected from the same person in different time, the identification is true.

According to Table 2, the weighted voting scheme has a good performance in identification experiments. And all the remaining 20 sets of recorded data are tested and correctly identified.

As a further step, the weighted coefficient vectors are set to *W* = [0, 0, 0, 1], *W* = [1, 1, 1, 1], *W* = [1, 1, 1, 2], *W* = [2, 3, 1*,ω*_4_] and *W* = [2, 2, 1,*ω*_4_], respectively. Then the numbers of successfully recognized datasets are 23, 26, 27, 27 and 30, respectively. It is known that the weighted coefficients have great effects on identification performances. Among the weighted coefficients, the similarity degree of characteristic curves has the highest effect, but the identification cannot depend on this coefficient alone. For experiments conducted in this paper, the weighted coefficient vector *W* = [2, 2, 1,*ω*_4_] was adopted and resulted in an excellent identification performances, while the error of identification is zero.

## 6. Conclusions

The accelerometer and gyrometer integrated in smartphones provide users with a convenient platform for gait analysis. In this paper, a new method for gait analysis is proposed based on an iPhone. The Fourth-order Runge-Kutta algorithm and quaternion are applied to combine inertial data so as to solve linear acceleration and eliminate the errors caused by attitude change and gravitational acceleration. The gait characteristic parameters and characteristic curves are analyzed, then a weighted voting scheme is adopted for gait identification. Simulation and experiment results demonstrate good performance of the proposed scheme. In future study, the gait database will be improved to support gait identification under different scenarios, e.g., different motion status of tested subjects.

## Figures and Tables

**Table 1.** Indices for the database. D1∼D10 represent the 10 samples in the database. The units of gait frequency and dynamic range are Hz and m/s^2^, respectively.

**Indices**	**D1**	**D2**	**D3**	**D4**	**D5**	**D6**	**D7**	**D8**	**D9**	**D10**
Gait Frequency	1.036	1.041	0.957	1.009	0.939	0.845	1.002	0.945	0.987	1.019
Symmetry Coef.	0.534	0.563	0.571	0.404	0.669	0.525	0.623	0.597	0.576	0.914
Dynamic Range	26.480	29.301	25.300	22.379	24.891	25.618	27.051	20.674	21.567	14.736

**Table 2.** Voting results for 10 measured data. M1∼M10 represent the 10 measured data. D1∼D10 represent the 10 samples in the database. The number in the frame is the minimum number in each row. True/False represents the current identification is true or false.

**Data Name**	**D1**	**D2**	**D3**	**D4**	**D5**	**D6**	**D7**	**D8**	**D9**	**D10**	**True/False**
M1	$\begin{array}{c} 17 \end{array}$	26	48	53	50	71	61	61	36	72	True
M2	28	$\begin{array}{c} 17 \end{array}$	29	47	52	48	39	45	30	50	True
M3	50	65	$\begin{array}{c} 27 \end{array}$	71	45	68	55	31	45	93	True
M4	40	66	50	$\begin{array}{c} 16 \end{array}$	73	72	45	52	54	82	True
M5	51	70	54	57	$\begin{array}{c} 17 \end{array}$	77	46	39	43	96	True
M6	45	46	29	49	51	$\begin{array}{c} 12 \end{array}$	48	43	41	76	True
M7	51	58	65	44	65	87	$\begin{array}{c} 22 \end{array}$	50	36	72	True
M8	46	58	24	73	68	76	46	$\begin{array}{c} 20 \end{array}$	45	94	True
M9	48	45	53	45	79	72	69	48	$\begin{array}{c} 22 \end{array}$	69	True
M10	76	61	48	62	53	90	66	44	28	$\begin{array}{c} 22 \end{array}$	True
